# Formerly Incarcerated Community Health Workers Engaging Individuals Returning From Incarceration Into Primary Care: Results From the Transition Clinic Network

**DOI:** 10.3389/fpubh.2021.681128

**Published:** 2021-08-05

**Authors:** Jenerius A. Aminawung, Tyler D. Harvey, Jerry Smart, Joseph Calderon, Anna Steiner, Elizabeth Kroboth, Emily A. Wang, Shira Shavit

**Affiliations:** ^1^SEICHE Center for Health and Justice, General Internal Medicine, Yale School of Medicine, New Haven, CT, United States; ^2^San Francisco Public Health Foundation, San Francisco, CA, United States; ^3^Department of Family and Community Medicine, University of California, San Francisco, San Francisco, CA, United States

**Keywords:** community health worker, incarceration, reentry, primary care, care integration

## Abstract

Over half a million individuals return from United States prisons and millions more from jails every year, many of whom with complex health and social needs. Community health workers (CHWs) perform diverse roles to improve health outcomes in disadvantaged communities, but no studies have assessed their role as integrated members of a primary care team serving individuals returning from incarceration. Using data from participants who received primary care through the Transitions Clinic Network, a model of care that integrates CHWs with a lived experienced of incarceration into primary care teams, we characterized how CHWs address participant health and social needs during interactions outside of clinic visits for 6 months after participants established primary care. Among the 751 participants, 79% had one or more CHW interactions outside of the clinic documented. Participants with more comorbid conditions, longer stays during their most recent incarceration, and released with a prescription had more interactions with CHWs compared to those with fewer comorbidities, shorter stays, and no prescription at release. Median number of interactions was 4 (interquartile range, IQR 2–8) and 56% were in person. The most common issues addressed (34%) were social determinants of health, with the most common being housing (35%). CHWs working in interdisciplinary primary care teams caring for people with histories of incarceration perform a variety of functions for clients outside of scheduled primary care visits. To improve health outcomes among disadvantaged populations, CHWs should be able to work across multiple systems, with supervision and support for CHW activities both in the primary care clinic and within the community.

## Introduction

Over 600,000 individuals return home from prison each year in the United States (US), as it continues to grapple with mass incarceration (1). More return from jails, where about 160,000 individuals on any given day are serving sentences that are less than a year (2). Those incarcerated are disproportionately from poor and racial and ethnic minority populations. Individuals who have experienced incarceration have a higher prevalence of chronic disease conditions compared to their age-matched counterparts who have not experienced incarceration (3–5), with 8 in 10 men and 9 in 10 women in the reentry population reporting at least one chronic condition (6).

Upon release, many individuals return to their communities that are disproportionately experiencing health and social inequities. Returning from incarceration is associated with distinct challenges, such as barriers to securing housing and employment based on one's criminal record, reconnecting with family members, and avoiding reincarceration (7). Health care access is often episodic within the first year following release, mainly occurring through high-cost emergency department visits (8). People returning home from incarceration thus require a community-tailored approach to address the social determinants of health and improve management of chronic health conditions.

The Transitions Clinic Network (TCN) is a national consortium of 45 primary care centers that address the health and social needs of individuals returning from jail and prison (9). Patients are typically those older than 50 years of age or with chronic health conditions, such as physical health conditions (hepatitis C, hypertension, diabetes), mental health conditions (depression, post-traumatic stress disorder, schizophrenia), or substance use disorders (opioid use disorder). The crux of each TCN program are interdisciplinary primary care teams with community health workers (CHWs) with histories of incarceration, who are tasked with identifying and supporting patients returning home from incarceration who are at risk for poor health outcomes. TCN CHWs receive specialized training from the City College of San Francisco through the post-prison healthcare worker certification program administered by the City College of San Francisco to train and professionalize CHWs. The training focuses on the system of mass incarceration, the impacts of incarceration on the health and social needs of returning community members, and the roles of CHWs in providing patient-centered care to these individuals. In clinic, the CHWs use their personal experience of incarceration, their social networks, and awareness of the criminal legal system to bridge knowledge gaps and build therapeutic alliances between the healthcare team and patient populations with high levels of mistrust. They educate the healthcare team about patients' challenges, facilitate patient-provider communication, and help patients navigate and build trust in the medical system. As integrated members of a care team, TCN CHWs also provide essential input on the design of programs and services and advocate for changes in clinic policies and practices to create a more welcoming environment for patients with histories of incarceration.

In addition to supporting patients during clinic visits, TCN CHWs spend a comparable amount of time within the community, where they address social determinants of health, such as housing, food access, or employment, and link patients with community agencies. They play an imperative role as frontline health workers and connect with patients prior to or immediately after release from incarceration, as well as advocate on patients' behalf in interactions with the criminal legal system, especially courts, probation, and parole when appropriate. In general, CHWs provide health education, counseling, social support and advocacy (10, 11). TCN CHWs provide similar functions, guided by their unique understanding of how incarceration impacts chronic health management, and the specific barriers criminal records pose to obtaining housing, food, or employment. Though several studies have described the diverse roles of CHWs (12–14) in caring for disadvantaged patient populations, none have specifically detailed activities of CHWs caring for patients transitioning from incarceration.

Past studies show that receiving care in a TCN program improves primary care engagement and reduces emergency department utilization, preventable hospitalizations, length of hospital stay, and future contact with the criminal legal system (9, 15, 16) as compared to receiving care in standard primary care provider, thus isolating the impact of having a community health worker as part of the primary care team. Further, a qualitative study conducted by a TCN community health worker demonstrated that having a person who is formerly incarcerated serve as a community health worker was critical to patients' forging connections and building trust in the health care system (17). In this brief report, we describe the unique role played by community health workers during their interactions with patients just released from incarceration.

## Methods

### Setting and Participants

This study uses data collected on 751 participants that consented to provide information as part of a Center for Medicare and Medicaid Innovation (CMMI) sponsored healthcare innovation project across 11 TCN programs between May 2013 and February 2015 (9). Participants were recently released from prison (within 6 months) and had a diagnosed chronic health condition or were aged 50 years or more.

### Data Collection

Participants were administered a baseline questionnaire upon establishing care with a TCN program, which collected data on socio-demographics (age, race/ethnicity, gender, highest education attained), basic needs (housing, food security, and health insurance), medical history, self-reported health, health literacy, and engagement with the health system prior to their most recent incarceration. We grouped responses on housing status into four categories (homeless, transitional housing, staying with family/friends, renting, or owning), and health insurance was categorized into whether the participant had insurance or not (Yes/No). Food insecurity was assessed with the singular question “Have you gone a whole day without food since your release from prison/jail because you did not have enough money to feed yourself?” Participants were asked a list of chronic health conditions which we then categorized into three groups based on the number of reported health conditions—(1) no reported condition, (2) one to three conditions, and (3) four or more conditions. Self-reported general health was combined into good (Excellent/Very Good/Good) and poor (Fair/Poor) health. Health literacy was measured using The Newest Vital Sign (NVS) (18), a short valid assessment of literacy in primary care, and scores were also combined into adequate (score ≥4) and inadequate (score ≤3) literacy (19). We also collected information on participants' most recent incarceration, including when it started and when they were released, and if they were released with any prescriptions.

### CHW Activity Log

As part of the study, CHWs were required to document all closed-loop interactions (two-way communication) with participants that occurred outside of a scheduled clinic visit, using a CHW encounter form. (CHWs were present at every clinic visit and were not asked to document that interaction.) CHWs collected information on the date of the encounter, who initiated the interaction (participant or CHW), mode of communication (in-person, phone, or electronic), purpose/type of interaction, issue(s) addressed during the interaction, and amount of time that the CHW spent with the participant during each interaction.

### Data Analysis

First, we compared participants with and without any documented CHW interaction outside of the clinic by sociodemographic and reported health characteristics. Among those with documented CHW interactions outside of the clinic, we characterize the interactions using descriptive statistics, including mode of communication, and purpose of interaction and issues addressed. Specifically, the issues addressed by the CHW were grouped into the following categories: physical health management (medications, physical health, care coordination into physical health management), behavioral health management (crisis management, mental health, and substance use), and social determinants of health (housing, employment, etc.). The study was approved by the Yale University School of Medicine Human Investigation Committee, and the Office for Human Research Protections in the US Department of Health and Human Services. All analyses were conducted using SPSS version 26.

## Results

Of the 751 participants, 547 (79%) had at least one documented interaction with a CHW outside of a primary care visit within 6 months of establishing care. Participants with at least one CHW interaction were older, mean age 47 (±11.0) years, compared to 44 (±11.5) years for those without a CHW interaction. Both groups were not significantly different in terms of gender, race, ethnicity, and education (Table 1). Participants who reported housing issues, food insecurity, not having primary care before incarceration, or lack of insurance were equally likely to have a CHW interaction, as those able to meet these basic needs. Those with four or more comorbid conditions (48 vs. 32%), released from incarceration with a prescription (78 vs. 70%), and a longer length of stay during their most recent incarceration (IQR 16–108 vs. 12–71) were more likely to have a CHW encounter (Table 1).

**Table 1:** Participant characteristics and CHW interactions outside of the primary care clinic (*N* = 751).

	**No CHW interaction (*N* = 204)**	**Had one or more CHW interaction (*N* = 547)**	***P*-value**
Characteristic	*N* (%)	*N* (%)	
Age, years; mean (±SD)	43.5 (±11.5)	47.1 (±11.0)	<0.001
**Gender**
Female	27 (13.2)	84 (15.4)	0.49
Male	177 (86.8)	463 (84.6)	
**Race/Ethnicity**
Non-Hispanic White	36 (17.6)	98 (17.9)	0.70
Non-Hispanic Black	91 (44.6)	261 (47.7)	
Hispanic	68 (33.3)	159 (29.1)	
Other	9 (4.4)	29 (5.3)	
**Highest education level**
Less than high school	122 (60.1)	318 (58.7)	0.71
High school graduate	35 (17.2)	86 (15.9)	
Some college/graduate	46 (22.7)	138 (25.5)	
**Housing status at first clinic visit**
Homeless	53 (26.0)	132 (24.1)	0.67
Transitional housing	72 (35.3)	210 (38.4)	
Family/friends	55 (27.0)	154 (28.2)	
Rent/own	24 (11.8)	51 (9.3)	
Had gone 24 h without food	34 (16.8)	117 (21.5)	0.18
Inadequate health literacy^*^	113 (59.5)	246 (52.9)	0.14
No health insurance at first visit	83 (40.9)	218 (39.9)	0.80
**Self-reported general health**
Fair/poor	85 (41.9)	261 (47.8)	0.16
Good/very good/excellent	118 (58.1)	285 (52.2)	
**Comorbid conditions**
None	31 (15.2)	80 (14.6)	<0.001
1 to 3	108 (52.9)	202 (36.9)	
4 or more	65 (31.9)	265 (48.4)	
Released with medication prescription	142 (69.6)	427 (78.1)	0.02
No established primary care before incarceration	91 (44.8)	262 (48.4)	0.41
Length of last incarceration, months; median (IQR)	39 (12–71)	39 (16–107.5)	0.04^¶^

* *Health literacy values only for those that provide enough responses to be able to compute a score based on the scale scoring algorithm (655 of 751 participants)*.

¶ *Mann Whitney U test p-value. SD, standard deviation; IQR, interquartile range*.

Overall, there were 3,342 documented interactions, with a median of 4 (IQR, 2–8) interactions per participant among those with at least one CHW interaction outside of a scheduled primary care visit. About half of the interactions were initiated by the CHW and the median time the CHW spent with the participant during an interaction was 23 min (IQR, 17–34). Most of the interactions were in person (56%), and CHWs addressed a range of concerns including preparing or assisting participant before a scheduled health care visit (48%), following up with participant after a visit (41%), and addressing social determinants of health (36%) (Table 2).

**Table 2:** Characteristics of CHW encounters within 6 months of establishing care with the TCN (*N* = 547).

**Characteristic**	***N***	**%**
Total	3,342	100.0
Median number (IQR)	4 (2–8)	
Median duration, minutes (IQR)	23 (17–34)	
**Initiated contact**
Patient	1,723	51.6
CHW	1,619	48.4
**Mode of interaction**
In person	1,867	55.9
Telephone	1,288	38.5
Text messages	177	5.3
Email	10	0.3
**Timing of encounter**
Before primary care visit	1,618	48.4
Post primary care visit	1,379	41.3
Post ED/hospitalization	40	1.2
Post criminal justice system contact	13	0.4
Accompanied patient to a service	292	8.7
**Concerns addressed** ^**¶**^
Social determinants of health	1,200	35.9
Medication management	446	13.3
Physical health	347	10.4
Care coordination	515	15.4
Mental/behavioral health	166	5.0
Other (e.g., emotional support, wellness check-in)	495	14.8

¶ *Percentages add up to over 100% because more than one concern was addressed at some interactions. IQR, interquartile range*.

**Figure 1:**
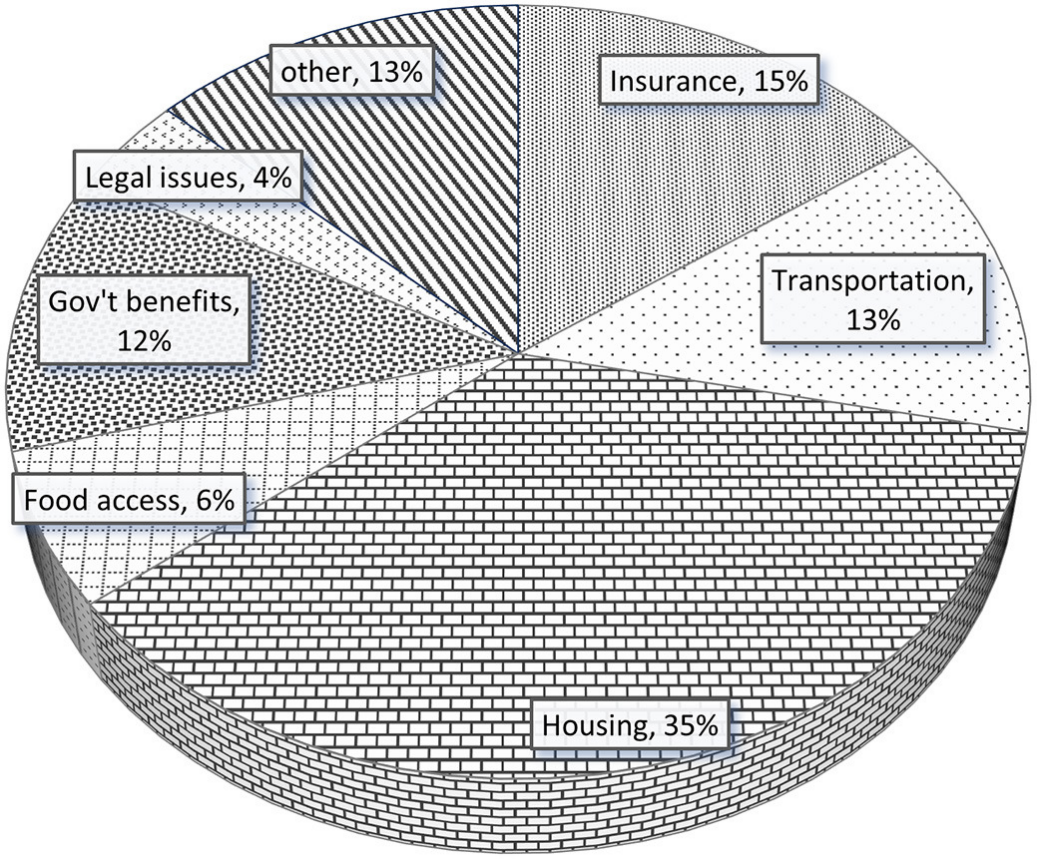
Social determinants of health addressed during CHW interactions.

**Figure 2:**
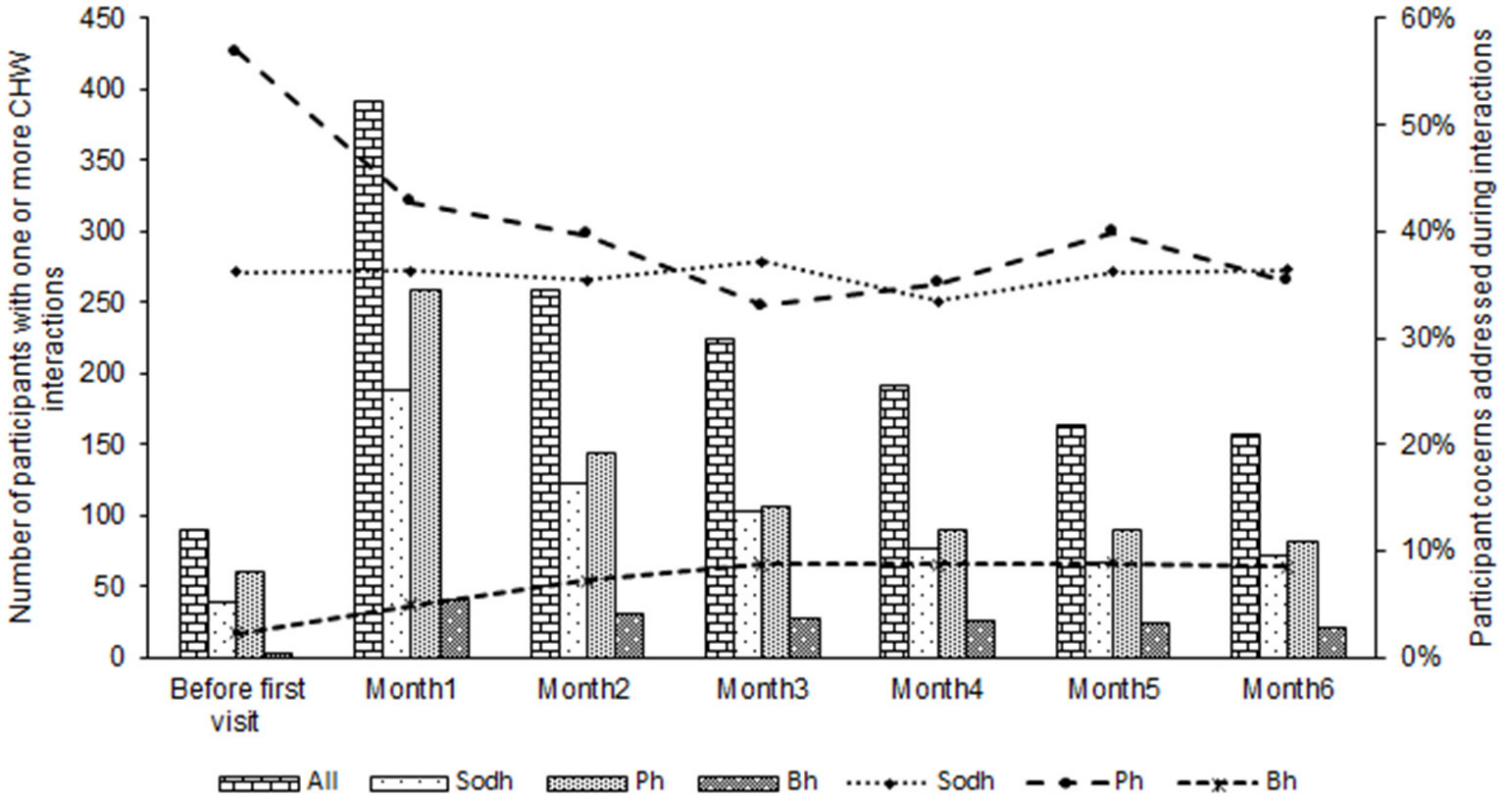
Number of participants with a CHW interaction and participant issues addressed during interactions over time. NB: Bars represent number participants with an interaction and issues addressed. Lines represent proportion of all interactions that addressed Sdoh, Bh, and Ph issues. Sdoh, social determinants of health, Ph, physical health management; Bh, behavioral health management. Some participants had more than one concern address during an interaction with the CHW.

For interactions that addressed social determinants of health, the most common issue addressed was housing (35%), followed by health insurance (15%), transportation (13%), and accessing government benefits (12%) (Figure 1). Figure 2 illustrates how the total number of participants with a CHW interaction and proportion of participant concerns addressed among all interactions changed over time. Sixteen percent of participants with CHW interactions had an interaction between release from prison and their first clinic visit, and most interactions occurred within 1 month after establishing care within the clinic.

## Discussion

Our characterization of the interactions between CHWs with lived experience of incarceration and patients returning from incarceration outside of the primary care setting illustrates the multi-faceted roles played by CHWs as they assisted their clients in improving their health and successfully reentering into the community. Participants who were older, those with more medical needs (had more co-morbidities or had a prescription that required refill), and those more disconnected from the community (had longer prison stay) were more likely to have a CHW encounter.

CHWs engaged with participants even before their first primary care visit, and most of the interactions registered were within the first month after establishing care. Most interactions were in-person and occurred around scheduled primary care visits, with 48% before and 41% after a primary care visit, and addressed a variety of patient needs, including social determinants of health, medications management, care coordination and physical and behavioral health coaching. It is worth noting that all interactions before the first primary care visit occurred in the community after release from prison due to barriers related to prison in-reach at the time of this study. To optimize engagement in primary care services and connection to community resources at reentry, CHWs should be allowed to interact with individuals prior to release from incarceration. These interactions may take the form of one-on-one in-person meetings between CHWs and patients, phone, or video calls, and in-person presentations to groups of individuals nearing release. This shift in paradigm would require a re-alignment of Medicaid payment structures to cover CHW activities, such as allowing Medicaid enrollment and reimbursement 30 days pre-release as seen in some states (20).

Among social determinants of health addressed during interactions by CHWs in our study, assisting participants with housing (35%) was the number one issue. Individuals who have experienced incarceration have high rates of unstable housing or homelessness that can negatively affect both reentry and health outcomes (21–23). This is driven by lack of social support, discriminatory policies and other collateral consequences of incarceration that makes it difficult to access stable housing and is accentuated in the immediate post-release period (23, 24). As community health workers within the TCN were especially attuned to the structural barriers to housing following release, it is not surprising that assistance with housing was the primary social issue addressed by CHWs in our study.

Individuals returning from incarceration have complex needs spanning social determinants of health, physical and behavioral health. However, the provision of post-release support to meet these needs is often inadequate because it fails to appreciate the unique difficulties patients encounter and the complexity of health care and social service systems (25). The TCN model encompasses aspects from three common CHW care models described in the US: extension of clinic systems, CHWs working through community non-profits and CHWs at the interface of health systems and the community (26). TCN CHWs' worked across systems performing multiple functions such as care coordination, health coaching, social support, resource linkages, case management, medication management, advocacy, and follow-up among other responsibilities. By integrating CHWs fully into primary care teams but also enabling CHWs the latitude to provide support and services within the community, CHWs are more able to help patients prioritize the many competing priorities of reentry from correctional systems.

## Limitations

While our study is unique in reporting on the role and functions of CHWs with lived experience of incarceration employed by TCN primary care programs, there are some limitations. The current study does not capture the full range of functions performed by TCN CHWs, as we only collected data on CHW interactions outside of the scheduled primary clinic visit. Data on CHW interactions during primary health care clinic visits—and activities not linked to individual patients such as policy advocacy—could elucidate a different set of roles played by CHWs. Also, CHWs collected data on paper forms while in the community and later entered this information into an electronic data collection platform, which could have led to some underreporting if the CHW was not in possession of a form at the time of interaction. Last, data were collected and reported by CHWs based on their assessment of patient needs and some mislabeling of patient concerns may have occurred.

## Conclusion

Individuals returning from incarceration have complex medical and social service needs. To address these needs, CHWs must be able to work across multiple systems and perform a broad range of functions, including assisting individuals with navigating health, social service, and criminal legal systems. Improved tracking of CHWs' activities through mobile electronic format (i.e., smartphone app) that can be linked to the electronic health records would not only enhance care management of people returning from incarceration, CHW supervision and support, but could be helpful for illuminating the full range of activities CHWs participate in (during clinic and outside in the community), facilitating patient outcome evaluation and developing reimbursement structures for CHW activities. Further, establishing standardized quality metrics for primary care delivery for this vulnerable population including CHW activities are needed and should address the full range of patients' health and social service needs.

## Data Availability Statement

The raw data supporting the conclusions of this article is available from the corresponding author, on reasonable request.

## Ethics Statement

The studies involving human participants were reviewed and approved by Yale University School of Medicine Human Investigation Committee, and the Office for Human Research Protections in the US Department of Health and Human Services. The patients/participants provided their written informed consent to participate in this study.

## Author Contributions

JA: study conception and design, data analysis, writing original draft, and writing review and editing. JS and JC: data collection, review, and editing. TH, AS, and EK: writing—review and editing. EW and SS: study conception and design, funding acquisition, and writing review and editing. All authors contributed to the article and approved the submitted version.

## Author Disclaimer

The contents of this article are solely the responsibility of the authors and do not necessarily represent the official views of the Department of Health and Human Services or any of its agencies.

## Conflict of Interest

The authors declare that the research was conducted in the absence of any commercial or financial relationships that could be construed as a potential conflict of interest.

## Publisher's Note

All claims expressed in this article are solely those of the authors and do not necessarily represent those of their affiliated organizations, or those of the publisher, the editors and the reviewers. Any product that may be evaluated in this article, or claim that may be made by its manufacturer, is not guaranteed or endorsed by the publisher.
